# Cold weather and primary monosymptomatic enuresis

**DOI:** 10.1590/S1677-5538.IBJU.2021.0236.1

**Published:** 2021-11-30

**Authors:** José Murillo B., José de Bessa

**Affiliations:** 1 Universidade Federal de Juiz de Fora Hospital Universitário Serviço de Urologia Pediátrica e Divisão de Urologia Juiz de For a MG Brasil Serviço de Urologia Pediátrica e Divisão de Urologia do Hospital Universitário da Universidade Federal de Juiz de Fora – UFJF, Juiz de For a, MG, Brasil; 2 Hospital e Maternidade Therezinha de Jesus Faculdade de Ciência Médicas e da Saúde de Juiz de Fora MG Brasil Hospital e Maternidade Therezinha de Jesus da Faculdade de Ciência Médicas e da Saúde de Juiz de Fora (SUPREMA), MG, Brasil; 3 Universidade Estadual de Feira de Santana Departamento de Urologia Feira de Santana BA Brasil Departamento de Urologia, Universidade Estadual de Feira de Santana, UEFS, Feira de Santana, BA, Brasil

## COMMENT

This is an interesting article addressing the effects of cold (winter season) in treatment results of primary monosymptomatic enuresis (PME). Although retrospective, the study includes a fair number (393) of children that were treated with desmopressin in a subtropical climate region of China, and it uses the International Children's Continence Society Criteria to define their results (1).

Although in our clinical daily practice we observe, and also hear from parents, that children presenting PME does worse during the wintertime, few studies have described this relationship between cold months and worsening or greater difficulty in treating enuresis. Previously, Tas et al., 2014, have shown that the number of wet nights per month and the number of enuretic episodes for one night increased during the winter months, which negatively impacts in the quality of life of these children in this period of the year (2).

One explanation for this association could be increased urine production during the cold period due to reduced loss of water by sweating despite reduced intake. It is known, based on experimental studies in rats, that lower temperatures reduce the difference between water intake and urine output significantly, as well as renal concentrating responses, (3) anti-diuretic hormone (ADH) secretion is also decreased (4).

These mechanisms may explain an increase in nocturnal urine output during the cold months of the year but no study was found that definitely explain or correlate cold weather to arousal or wakening problems, which is one of the main mechanisms related to the cause of enuresis up to today.

The authors have evaluated other important factor associated with enuresis in their study, such as severity of symptoms, family history of enuresis, obesity, sleep quality (snoring), among others, and in a multivariate analysis found only severity of symptoms and winter season as factors related to treatment failure with the use of desmopressin in children presenting PME (5). Similar results were found by Shiroyanagi et al., 2014, evaluating the use of enuresis alarm during wintertime. In their study, initiating treatment with enuresis alarm during winter season was an independent risk factor with 3.13 more chance of treatment failure (6).

As we all know, desmopressin and enuresis alarm have different mechanisms of action and are indicated for enuresis associated with nocturnal polyuria or not, respectively, and each treatment seems to go worse during winter season. These poor results during the cold months of the year with two different treatment modalities lead us to think that cold may also influence sleep quality and arousal of these children. It has been shown that apnea-hypopnea index has an inverse correlation to temperature (7), and that sleeping problems (difficulty initiating sleep, difficulty maintaining sleep, and excessive daytime sleepiness) occur more frequently in winter season (8).

All this raise the question whether the worst results in treating enuresis during winter season with desmopressin, presented by Sun et al., are related to changes in nocturnal urinary production, changes in sleep pattern or both. This is an open field for new studies and, as we know, enuresis etiology is multifactorial and cold weather may also play a rule in this already complicated and difficult to understand problem that affects millions of children worldwide.
